# Coexistence of Primary Colorectal Cancer and Multiple Colonic Metastases from Pancreatic Tail Cancer: A Case Report

**DOI:** 10.70352/scrj.cr.25-0828

**Published:** 2026-05-13

**Authors:** Marina Uemura, Yoshihiro Takashima, Yoshinori Munemoto, Susumu Amaya, Takuro Terada, Kenichiro Saito, Masanari Shimada, Sho Yamada, Tatsuhiko Kohno, Toshiki Sakaguchi, Yuie Katsuyama, Renta Kobori, Kodai Hasegawa, Yasuni Nakanuma

**Affiliations:** 1 Department of Surgery, Fukui-ken Saiseikai Hospital, Fukui, Fukui, Japan; 2 Department of Diagnostic Pathology, Fukui-ken Saiseikai Hospital, Fukui, Fukui, Japan

**Keywords:** pancreatic cancer, colonic metastasis, colorectal cancer, immunohistochemistry, carbohydrate antigen 19-9 (CA19-9)

## Abstract

**INTRODUCTION:**

Colonic metastasis from pancreatic cancer is extremely rare. Distinguishing metastatic pancreatic cancer from primary colorectal cancer is clinically important because the therapeutic strategies differ substantially. We report an exceptionally rare case of pancreatic tail cancer with multiple colonic metastases coexisting with primary colorectal cancer.

**CASE PRESENTATION:**

An 83-year-old man presented with right lower abdominal pain, constipation, and weight loss. Four years earlier, MRI and magnetic resonance cholangiopancreatography (MRCP) had demonstrated a cystic lesion in the pancreatic tail. On admission, contrast-enhanced CT revealed a pancreatic tail tumor with suspected invasion of adjacent organs, lesions in the ascending and sigmoid colon, para-aortic lymph node metastasis, and peritoneal dissemination. Barium enema and colonoscopy showed stenotic lesions, and bowel obstruction developed. Palliative open right hemicolectomy and sigmoid colectomy were therefore performed. Gross examination revealed a mucosa-based type 2 lesion in the ascending colon and separate serosa-predominant lesions in the ascending and sigmoid colon. Histopathological and immunohistochemical analyses showed that the type 2 lesion represented primary colorectal cancer, whereas the other colonic lesions and peritoneal nodules were metastatic pancreatic cancer. The patient developed postoperative ileus and pneumonia, was discharged on POD 24, and died 6 months after surgery.

**CONCLUSIONS:**

Colonic metastasis from pancreatic cancer may sometimes be difficult to distinguish from primary colorectal cancer. In patients with pancreatic cancer, colonic lesions with relatively preserved mucosa and predominant serosal involvement should be considered in the differential diagnosis of metastatic disease. Markedly elevated carbohydrate antigen 19-9 levels may also provide an additional clue to pancreatic origin, although they are not diagnostic. Careful integrated assessment of the clinical course, imaging, and pathological findings is essential for accurate diagnosis and appropriate management.

## Abbreviations

CA19-9
: carbohydrate antigen 19-9
CDX2
: caudal type homeobox 2
CK
: cytokeratin
IPMN
: intraductal papillary mucinous neoplasm
MRCP
: magnetic resonance cholangiopancreatography
SATB2
: special AT-rich sequence-binding protein 2
SUVmax
: maximum standardized uptake value

## INTRODUCTION

Pancreatic cancer is frequently diagnosed at an advanced stage and commonly metastasizes to the liver, lungs, peritoneum, and lymph nodes.^1)^ In contrast, metastasis to the colon is extremely rare. When colonic lesions are detected in patients with pancreatic cancer, distinguishing metastatic disease from primary colorectal cancer is clinically important because therapeutic strategies differ substantially. Only a limited number of cases of colonic metastasis from pancreatic cancer have been reported, and, to our knowledge, no previous reports have described the simultaneous coexistence of primary colorectal cancer and multiple colonic metastases derived from pancreatic cancer. We herein report such an exceptionally rare case and discuss the relevant diagnostic and therapeutic considerations.

## CASE PRESENTATION

**Fig. 1:**
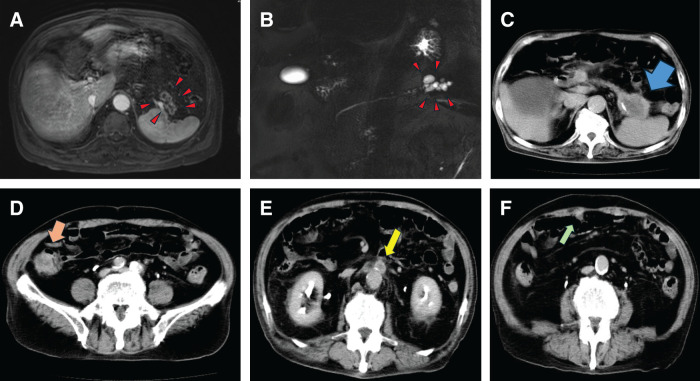
Radiological findings. (**A**) MRI (e-THRIVE) obtained 4 years before presentation demonstrates a cystic lesion in the pancreatic tail (red arrowheads). (**B**) MRCP obtained 4 years before presentation shows the cystic lesion in the pancreatic tail (red arrowheads). (**C**–**F**) Contrast-enhanced CT at presentation reveals a pancreatic tail tumor (blue arrow), colonic tumors (orange arrow), para-aortic lymph node metastasis (yellow arrow), and peritoneal dissemination (green arrow). e-THRIVE, enhanced T1-weighted high-resolution isotropic volume examination; MRCP, magnetic resonance cholangiopancreatography

**Fig. 2:**
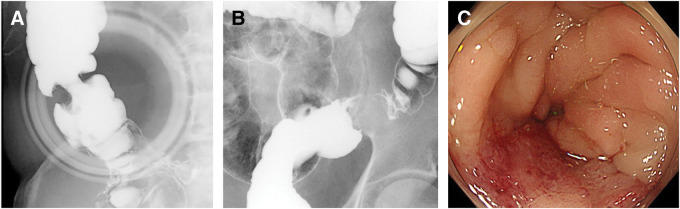
Barium enema and endoscopic findings. (**A**) Barium enema demonstrates an apple-core lesion in the ascending colon. (**B**) An elevated lesion causing asymmetric stenosis is observed in the sigmoid colon. (**C**) Colonoscopy reveals severe stenosis in the sigmoid colon, preventing passage of the endoscope.

An 83-year-old man presented with a 1-month history of right lower abdominal pain, constipation, and unintentional weight loss. He had a history of a pancreatic cystic lesion suggestive of IPMN, which had been incidentally detected 4 years earlier. Follow-up had been recommended at a local clinic, although regular surveillance did not appear to have been continued. He also had a history of surgery for perforated duodenal ulcer, as well as hypertension, diabetes mellitus, gastroesophageal reflux disease, benign prostatic hyperplasia, and overactive bladder. There was no remarkable family history. He had smoked 15 cigarettes per day for 10 years, starting at the age of 23, and consumed approximately 2 servings of sake daily, although his alcohol intake had recently decreased. A fecal occult blood test was positive, and fluoroscopic examination at a referring hospital suggested tumors in the ascending and sigmoid colon. He was referred to our institution, where colonoscopy revealed severe stenosis of the sigmoid colon near the sigmoid-descending junction, prompting emergency admission. A flat elevated lesion was also observed in the rectum; however, the biopsy showed no malignant findings. Laboratory tests on admission revealed a carcinoembryonic antigen level of 5.4 ng/mL and a markedly elevated CA 19-9 level of 19723 U/mL. Other laboratory findings were within normal limits. MRI, including enhanced T1-weighted high-resolution isotropic volume examination (e-THRIVE) imaging, and MRCP obtained 4 years before presentation had demonstrated a cystic lesion in the pancreatic tail (**Fig. 1A** and **1B**). On admission, contrast-enhanced CT revealed a 5-cm mass in the pancreatic tail with suspected invasion of the spleen, stomach, and left adrenal gland. An enlarged para-aortic lymph node measuring 17 mm was observed at the level of the renal artery bifurcation. Tumorous lesions were also identified in the ascending and sigmoid colon, and peritoneal dissemination was suspected (**Fig. 1C**–**1F**). PET–CT revealed increased fluorodeoxyglucose uptake at the periphery of the pancreatic tail mass (SUVmax 4.4). Additional uptake was detected in the ascending and sigmoid colon (SUVmax approximately 5–7), as well as in the abdominal wall, mesentery, liver, spleen, pancreas, and para-aortic lymph nodes. Barium enema examination demonstrated an apple-core lesion in the ascending colon and a well-demarcated elevated lesion measuring approximately 25 mm in the sigmoid colon, causing asymmetric stenosis (**Fig. 2A** and **2B**). Colonoscopy revealed severe stenosis in the sigmoid colon near the sigmoid-descending junction, which prevented further advancement of the endoscope (**Fig. 2C**).

Based on these findings, a preoperative diagnosis of ascending colon cancer, sigmoid colon cancer, and peritoneal dissemination was made. Because the patient developed bowel obstruction, surgical resection was performed for symptom relief. Right hemicolectomy, sigmoid colectomy, and resection of abdominal wall metastatic nodules were performed. Under general and epidural anesthesia, the operation was started in the lithotomy position. A midline laparotomy revealed adhesions between the greater omentum and the upper abdominal wall. Tumors were identified in the ascending colon, sigmoid colon, and ileocecal region, all of which had invaded the abdominal wall. The sigmoid colon tumor showed continuous extension into the mesentery, and a disseminated nodule was observed on the anterior rectal wall near the peritoneal reflection. Sigmoid colectomy was performed first. Because the tumor had invaded the retroperitoneum near the sigmoid-descending junction, the colon was mobilized with partial resection of the involved retroperitoneum. The affected bowel was resected, D1 lymph node dissection was performed, and reconstruction was achieved by functional end-to-end anastomosis. Subsequently, right hemicolectomy was performed. After ligation and division of the ileocolic vessels, the involved bowel was resected, D1 lymph node dissection was performed, and reconstruction was achieved by functional end-to-end anastomosis. After thorough irrigation of the operative field, a drain was placed, and the operation was completed.

**Fig. 3:**
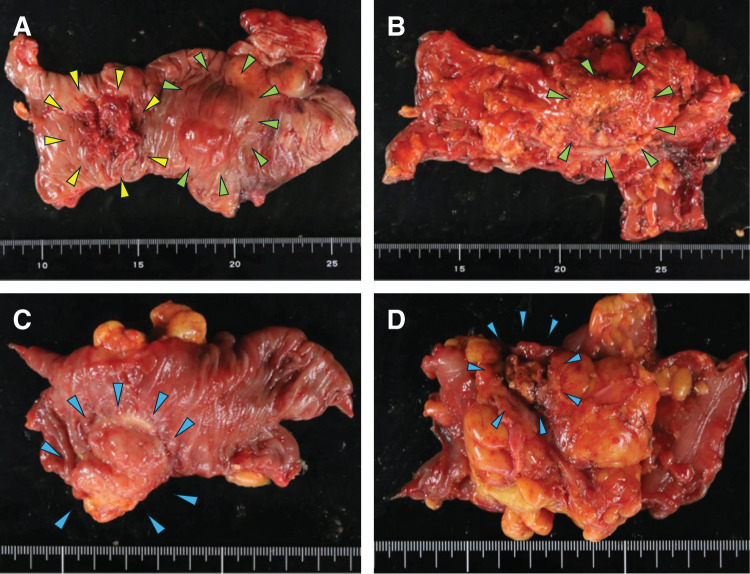
Gross findings of the resected specimen. (**A**, **B**) The ascending colon specimen shows a type 2 primary colorectal cancer (yellow arrowheads) and a separate serosa-predominant lesion suggestive of metastatic involvement (green arrowheads). (**C**, **D**) The sigmoid colon specimen shows a lesion mainly involving the serosal side, consistent with metastatic pancreatic cancer (blue arrowheads).

**Fig. 4:**
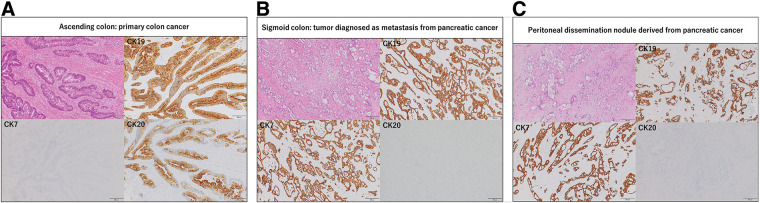
Histopathological and immunohistochemical findings. All histopathological and immunohistochemical panels are shown at the same magnification (×100), with consistent scale bars. (**A**) The type 2 lesion in the ascending colon shows well-differentiated tubular adenocarcinoma. Immunohistochemical analysis reveals negativity for CK7 and positivity for CK19 and CK20, consistent with primary colorectal cancer. (**B**) Immunohistochemical analysis of the sigmoid colon lesion shows positivity for CK7 and CK19 and negativity for CK20, suggesting metastasis from pancreatic cancer. (**C**) Immunohistochemical analysis of a peritoneal dissemination nodule shows positivity for CK7 and CK19 and negativity for CK20, supporting metastasis from pancreatic cancer. CK, cytokeratin

Gross examination of the resected specimens revealed a type 2 ulcerative lesion in the ascending colon, consistent with primary colorectal cancer, and separate serosa-predominant lesions in the ascending and sigmoid colon (**Fig. 3**). The metastatic lesions showed a submucosal tumor-like appearance with relatively preserved mucosal surfaces, in contrast to the mucosa-based primary colorectal lesion. Histopathological examination of the ascending colon revealed a type 2 ulcerative tumor measuring 2.8 × 2.8 cm and a separate submucosal tumor-like lesion measuring 2.2 × 4.2 cm. The type 2 lesion was diagnosed as well-differentiated tubular adenocarcinoma invading the subserosa and showed a CK7-negative/CK19-positive/CK20-positive immunophenotype, consistent with primary colorectal cancer. Metastases were identified in 2 of 10 dissected lymph nodes, both exhibiting an intestinal phenotype. In contrast, the submucosal tumor-like lesion in the ascending colon consisted of tubular adenocarcinoma predominantly located on the serosal side with moderate fibrous proliferation. This lesion showed positivity for CK7 and CK19 and negativity for CK20, indicating metastasis from pancreatic cancer. A similar semicircumferential submucosal tumor-like lesion measuring 2.8 × 2.8 cm was identified in the sigmoid colon, with identical histological and immunohistochemical features. Peritoneal and abdominal wall nodules also showed the same characteristics and were diagnosed as metastatic pancreatic cancer (**Fig. 4**).

Postoperatively, the patient developed ileus and pneumonia but was discharged on POD 24. One month later, he was readmitted with aspiration pneumonia. Considering his overall condition, chemotherapy was not administered, and palliative care was initiated. The patient died 6 months after surgery.

## DISCUSSION

Colonic metastasis from pancreatic cancer is extremely rare. To our knowledge, only 13 cases have previously been reported in the literature, and the present case represents the 14th reported case (**Table 1**).^2–14)^ Moreover, the coexistence of primary colorectal cancer and multiple colonic metastases derived from pancreatic cancer, as observed in the present case, has not been previously reported. Such a clinical presentation makes accurate diagnosis particularly challenging.

**Table 1:** Reported cases of colonic metastasis from pancreatic cancer

Author (year)	Age/sex	Timing	Primary site of pancreatic cancer	Colonic metastatic site	Preoperative diagnosis	CA19-9 (U/mL)	Treatment	Outcome	Immunohistochemistry
Bellows (2009)^2)^	45/M	Synchronous	Unknown	Ascending colon	Not diagnosed	Unknown	Surgery	Unknown	CK7−/CK20−
Ogu (2012)^3)^	85/F	Metachronous (2 y)	Pancreatic head	Sigmoid colon	Not diagnosed	Unknown	Surgery	Unknown	CK7 strong+/CK20 weak+
Inada (2013)^4)^	62/M	Metachronous (7 y 2 mo)	Pancreatic head	Ascending colon	Not diagnosed	1886.60	Surgery + gemcitabine	Died at 14 mo	CK7+/CK20−
Kim (2015)^5)^	64/M	Metachronous (1 y 11 mo)	Pancreatic tail	Cecum	Not diagnosed	5133.00	Surgery + gemcitabine	Alive at 6 mo	CK7 strong+
Kelley (2016)^6)^	67/F	Synchronous	Pancreatic tail	Sigmoid colon	Not diagnosed	Unknown	Surgery + FOLFIRINOX	Unknown	Unknown
Akatsuka (2017)^7)^	80/F	Synchronous	Pancreatic body	Sigmoid colon	Not diagnosed	Unknown	Surgery + gemcitabine	Died at 7 mo	CK7+/CK20−
Park (2019)^8)^	73/F	Synchronous	Pancreatic body	Sigmoid colon	Not diagnosed	Unknown	Surgery	Died at 7 mo	CK7 strong+/CK20 focal+
Nogueira (2018)^9)^	60/M	Synchronous	Pancreatic tail	Sigmoid colon	Diagnosed	60333.50	Surgery + FOLFOX	Died at 9 mo	CK7 +/CK20−
Kahl (2019)^10)^	91/F	Synchronous	Pancreatic head	Sigmoid colon	Not diagnosed	7480	Surgery	Unknown	CK7 +/CK20−
Ardalan (2022)^11)^	66/F	Synchronous	Pancreatic tail	Sigmoid colon	Not diagnosed	Unknown	Surgery + FOLFIRINOX	Died at 9 mo	CK7 +/CK20−
Kuroki (2022)^12)^	73/M	Metachronous (2 y 10 mo)	Pancreatic tail	Ascending colon	Not diagnosed	96 → 2634	Surgery	Alive at 2 y 9 mo	CK7 +/CK20−
Pacheco (2023)^13)^	78/M	Synchronous	Pancreatic body	Sigmoid colon	Not diagnosed	112444	Surgery	Unknown	Unknown
Meng (2023)^14)^	65/M	Synchronous	Pancreatic tail	Sigmoid colon	Diagnosed	2457	Gemcitabine + nab-paclitaxel	Unknown	CK7 strong+/CK20−
Present case (2025)	83/M	Synchronous	Pancreatic tail	Ascending and sigmoid colon	Not diagnosed	19723	Surgery	Died at 6 mo	CK7+/CK20−

CA19-9, carbohydrate antigen 19-9; CK, cytokeratin; F, female; M, male; mo, months; y, years

Preoperative diagnosis of colonic metastasis from pancreatic cancer is particularly difficult. Among the previously reported cases, only 2 were definitively diagnosed before surgery.^9,14)^ Diagnostic difficulty is even greater in metachronous cases, as colonic metastasis may occur long after initial pancreatic resection; a case occurring 7 years after pancreatic surgery has been reported.^4)^ Radiological modalities such as CT and MRI are insufficient to reliably distinguish metastatic lesions from primary colorectal cancer. Furthermore, metastatic lesions often predominantly involve the submucosa or serosal layer, making definitive diagnosis by endoscopic biopsy difficult. Malignant findings may not be detected on biopsy in some cases.^7,12)^ Even when malignant cells are obtained, the lesion may be diagnosed only as poorly differentiated adenocarcinoma^2)^ or a similar histologic type, without identification of the primary origin. Accurate differentiation between metastatic pancreatic cancer and primary colorectal cancer is clinically important because the treatment strategy differs substantially between these 2 conditions. Given this diagnostic difficulty, tumor markers may provide supportive information when considering the differential diagnosis. Previous reports have shown that many patients with colonic metastasis from pancreatic cancer exhibit markedly elevated CA19-9 levels. In general, CA19-9 is more likely to be elevated in pancreatic cancer than in primary colorectal cancer, particularly in advanced disease with distant metastasis.^15–17)^ In the present case, the CA19-9 level was also markedly elevated. However, CA19-9 should be interpreted with caution because it does not increase in Lewis antigen-negative individuals and may also be elevated in other benign or malignant conditions.

Immunohistochemical analysis plays a central role in distinguishing metastatic pancreatic cancer from primary colorectal cancer. Primary colorectal cancer typically shows a CK7-negative and CK20-positive immunophenotype, whereas pancreatic ductal adenocarcinoma is generally CK7-positive and CK20-negative.^18)^ In addition, SATB2 and CDX2 are useful markers of colorectal origin and may improve diagnostic accuracy when included in the immunohistochemical panel.^19,20)^ In the present case, although SATB2 and CDX2 were not examined, the metastatic lesions showed submucosal tumor-like morphology with minimal mucosal involvement and predominant serosal extension. These lesions were also positive for CK7 and CK19 and negative for CK20, whereas the primary ascending colon lesion showed conventional mucosa-based morphology and an intestinal immunophenotype. Therefore, in the overall clinicopathological context, the combination of morphological findings and the immunohistochemical results obtained in the present case was considered acceptable for diagnosis. However, because pancreatic cancer may occasionally show CK20 positivity, differentiation from primary colorectal cancer may be difficult when relying only on CK7/CK19/CK20 staining. In such cases, additional colorectal markers such as SATB2 and CDX2 may be useful.

When considering treatment, colonic metastasis from pancreatic cancer is regarded as distant metastatic disease, and systemic chemotherapy according to standard regimens for metastatic pancreatic cancer is often selected as the primary treatment. However, given the extremely limited number of reported cases, there is currently no established evidence regarding the optimal treatment strategy. In addition, bowel perforation due to tumor necrosis following chemotherapy has been reported,^9)^ indicating the need for careful monitoring during treatment. In the present case, open surgery was selected because bowel obstruction involved both the ascending and sigmoid colon, and preoperative endoscopic assessment of the proximal colon was limited by severe sigmoid stenosis, making endoscopic management such as stenting difficult. In addition, prior surgery for perforated duodenal ulcer and peritoneal dissemination suggested severe intra-abdominal adhesions, which made a laparoscopic approach less suitable. Proximal diversion would also likely have required ileostomy, raising concern about a high-output stoma, and postoperative stoma self-management was considered challenging because the patient and his wife were both in their 80s and lived alone as an elderly couple. Based on these considerations, open resection was considered the most reliable approach to relieve obstruction while maintaining intestinal continuity. On the other hand, depending on the level of obstruction, technical feasibility, and the patient’s overall condition, less invasive strategies such as colonic stenting, laparoscopic surgery, or stoma creation may also be considered.

The metastatic pathway from pancreatic cancer to the colon remains unclear, and both hematogenous spread and peritoneal dissemination have been proposed.^4,5,7)^ In the present case, the metastatic lesions were predominantly located on the serosal side of the colon and were accompanied by peritoneal and abdominal wall dissemination, suggesting implantation associated with peritoneal dissemination. In addition, our review of the previously reported cases, including the present case, showed a relatively frequent association with pancreatic body/tail tumors and sigmoid colon involvement. This distribution may be compatible with Meyers’ concept of intraperitoneal fluid circulation and preferential sites of tumor cell deposition.^21)^ In the present case, the invasive component showed a tubular adenocarcinoma pattern, and it remained unclear whether the tumor represented conventional pancreatic ductal adenocarcinoma or carcinoma derived from IPMN. If the tumor had been derived from intestinal-type IPMN, adaptation to a favorable microenvironment might be considered as a possible contributing factor to implantation and metastatic distribution. However, because the present tumor showed a tubular adenocarcinoma pattern, which is known to exhibit clinicopathological behavior similar to that of conventional pancreatic ductal adenocarcinoma, including prognosis and metastatic pattern, the unusual metastatic pattern could not be attributed specifically to an IPMN-derived background. Rather, the formation of the colonic lesions in this case may have been more strongly influenced by the aggressive invasive behavior shared by tubular adenocarcinoma of the pancreas. Because molecular analysis was not performed in this case, it remains unclear which factors promoted subserosal invasion and local proliferation, resulting in this rare metastatic pattern. Further accumulation of similar cases, together with molecular and genetic analyses, will be necessary to clarify the mechanisms underlying this unusual metastatic presentation.

## CONCLUSIONS

Colonic metastasis from pancreatic cancer is rare and may sometimes be difficult to distinguish from primary colorectal cancer. In patients with pancreatic cancer, metastatic disease should be included in the differential diagnosis when colonic lesions show relatively preserved mucosa and predominant serosal involvement. Markedly elevated CA19-9 levels may also provide an additional clue prompting consideration of pancreatic origin, although they are not diagnostic. Careful integrated assessment of the clinical course, imaging, and pathological findings, including repeated biopsy and expanded immunohistochemical analysis when necessary, is essential for accurate diagnosis and appropriate management.

## DECLARATIONS

### Availability of data and materials

The datasets generated and/or analyzed during the current study are available from the corresponding author on reasonable request.
